# Elucidating the Mechanisms of Sodium Benzoate in Alzheimer Disease: Insights from Quantitative Proteomics Analysis of Serum Samples

**DOI:** 10.1093/ijnp/pyad061

**Published:** 2023-10-24

**Authors:** Chieh-Hsin Lin, Hsin-Yi Liao, Hsien-Yuan Lane, Chao-Jung Chen

**Affiliations:** Department of Psychiatry, Kaohsiung Chang Gung Memorial Hospital, Chang Gung University College of Medicine, Kaohsiung, Taiwan; Graduate Institute of Biomedical Sciences, China Medical University, Taichung, Taiwan; School of Medicine, Chang Gung University, Taoyuan, Taiwan; Proteomics Core Laboratory, Department of Medical Research, China Medical University Hospital, Taichung, Taiwan; Graduate Institute of Biomedical Sciences, China Medical University, Taichung, Taiwan; Department of Psychiatry and Brain Disease Research Center, China Medical University Hospital, Taichung, Taiwan; Department of Psychology, College of Medical and Health Sciences, Asia University, Taichung, Taiwan; Proteomics Core Laboratory, Department of Medical Research, China Medical University Hospital, Taichung, Taiwan; Graduate Institute of Integrated Medicine, China Medical University, Taichung, Taiwan

**Keywords:** Alzheimer disease, D-amino acid oxidase, sodium benzoate, proteomics, label-free

## Abstract

**Background:**

N-methyl-D-aspartate receptors (NMDARs) are crucial components of brain function involved in memory and neurotransmission. Sodium benzoate is a promising NMDAR enhancer and has been proven to be a novel, safe, and efficient therapy for patients with Alzheimer disease (AD). However, in addition to the role of sodium benzoate as an NMDA enhancer, other mechanisms of sodium benzoate in treating AD are still unclear. To elucidate the potential mechanisms of sodium benzoate in Alzheimer disease, this study employed label-free quantitative proteomics to analyze serum samples from AD cohorts with and without sodium benzoate treatment.

**Methods:**

The serum proteins from each patient were separated into 24 fractions using an immobilized pH gradient, digested with trypsin, and then subjected to nanoLC‒MS/MS to analyze the proteome of all patients. The nanoLC‒MS/MS data were obtained with a label-free quantitative proteomic approach. Proteins with fold changes were analyzed with STRING and Cytoscape to find key protein networks/processes and hub proteins.

**Results:**

Our analysis identified 861 and 927 protein groups in the benzoate treatment cohort and the placebo cohort, respectively. The results demonstrated that sodium benzoate had the most significant effect on the complement and coagulation cascade pathways, amyloidosis disease, immune responses, and lipid metabolic processes. Moreover, Transthyretin, Fibrinogen alpha chain, Haptoglobin, Apolipoprotein B-100, Fibrinogen beta chain, Apolipoprotein E, and Alpha-1-acid glycoprotein 1 were identified as hub proteins in the protein‒protein interaction networks.

**Conclusions:**

These findings suggest that sodium benzoate may exert its influence on important pathways associated with AD, thus contributing to the improvement in the pathogenesis of the disease.

**Significance Statement**

Sodium benzoate has been proven to be a novel, safe, and efficient therapy for patients with Alzheimer disease (AD). However, in addition to the role of sodium benzoate as an NMDA enhancer, other mechanisms of sodium benzoate in treating AD are still unclear. Our proteome results demonstrated that sodium benzoate had the most significant effect on the complement and coagulation cascade pathways, amyloidosis disease, immune responses, and lipid metabolic processes. These findings suggest that sodium benzoate may exert its influence on important pathways associated with AD, thus contributing to the improvement in the pathogenesis of the disease.

## INTRODUCTION

Early intervention is the key for dementia treatment; however, the effectiveness and tolerability of acetylcholinesterase inhibitors (AChEIs), mainstream treatments for early-phase Alzheimer disease (AD), are still unsatisfactory (Birks, 2006; Burns et al., 2006). Anti-Aβ monoclonal antibodies are promising treatments for AD; however, they remain unable to halt AD progression (Soderberg et al., 2022). The lack of efficacy of AChEIs and immunotherapy against Aβ implies that there are other contributing factors to the development of AD, such as oxidative stress and dysfunction of N-methyl-D-aspartate receptors (NMDARs). Apart from AChEIs and Aβ antibodies, NMDAR antagonists have been developed to treat the middle-late phase of AD according to the glutamate excitotoxicity theory. However, a weak, partial, uncompetitive NMDAR antagonist has been approved for moderate-severe AD but exhibits limited efficacy in the early phase (Schneider et al., 2011). Studies have found that the density of NMDARs declines with age (Segovia et al., 2001), and D-serine, an NMDAR coagonist, decreases in the serum of AD patients (Hashimoto et al., 2004). NMDAR dysfunction may cause oxidative stress and downregulation of NMDARs (Hardingham and Bading, 2010).

Sodium benzoate, a D-amino acid oxidase inhibitor that is a potential indirect NMDAR enhancer, was reported to reverse NMDAR deficiency–mediated animal behavior (Matsuura et al., 2015). In addition, it may play a neuroprotective role by reducing oxidative stress, altering sex hormones, and having other routes (Xu et al., 2019; Lin et al., 2021). Sodium benzoate has been reported to improve the cognitive function of patients with AD in randomized, double-blind, placebo-controlled trials (Lin et al., 2014; Lane et al., 2023). In addition to the role of an NMDAR enhancer, other possible mechanisms of sodium benzoate in improving AD deserve further study.

Nanoflow liquid chromatography-mass spectrometry (nanoLC-MS)-based proteomics has been widely used to analyze the global protein content of a biological sample (Boja et al., 2011). By studying proteomics, the possible functions, roles, and interactions of proteins can be revealed, thereby being beneficial for elucidating disease mechanisms (Chang et al., 2018), discovering protein markers (Chang et al., 2018; Chen et al., 2021), and developing new drugs (Varshney and Mishra, 2023). Blood is an attractive sample for proteomic analysis because it reflects the overall systemic state of an organism and contains proteins derived from various tissues and organs. By studying the proteome of serum, we can gain insights into disease biomarkers, identify potential therapeutic targets, and understand disease mechanisms (Surinova et al., 2011). However, serum contains a wide range of protein concentrations, spanning several orders of magnitude. High-abundance proteins, such as albumin and immunoglobulins, can mask the detection of low-abundance proteins, which may be biologically relevant. Overcoming this dynamic range is a significant technical challenge in serum proteomics (Zhang et al., 2011).

In the current study, to uncover possible mechanisms of sodium benzoate in treating AD, the serum of AD patients before and after treatment with sodium benzoate and placebo was collected and analyzed with quantitative proteomics. To obtain a more thorough proteome profile, the serum proteins of each patient sample were fractionated by the off-gel method, an isoelectric-focusing fractionation method that enables the separation of proteins in a solution placed on an immobilized pH gradient (IPG) gel (Moreda-Pineiro et al., 2014; Fuente-Garcia et al., 2019). The serum proteins were separated into 24 subfractions by electrophoresis, and then each subfraction was individually trypsin-digested and subjected to nanoLC‒MS/MS analysis to collect each patient’s proteome profile. The proteome profiling of all patients was further compared with identify differentially expressed proteins. The serum proteomic results showed that sodium benzoate could affect some important pathways that play key roles in the pathogenesis of AD.

## METHODS

This was a subgroup analysis of a previous randomized, double-masked, placebo-controlled, 24-week trial conducted in Taiwan (Lin et al., 2014), which was approved by the institutional review board, registered with ClinicalTrials.gov (NCT01600469), and conducted in accordance with the current revision of the Declaration of Helsinki.

Among the 60 patients in the previous study (Lin et al., 2014), 13 patients with mild AD (6 sodium benzoate-treated patients and 7 placebo-recipients, who were matched in age and sex) were selected for the current study (Table 1). The laboratory analysis was conducted after the selection of the 13 participants and under blindness to the treatment group.

**Table 1.** Demographic and clinical characteristics of patients with mild Alzheimer disease

Demographics	Treatment groups		*P* value
	Sodium benzoate	Placebo	
	(n = 6)	(n = 7)	
Sex, female, n (%)	3 (50.0)	4 (57.1)	1.000^a^
Age, y, mean (SD)	73.5 (7.3)	67.6 (7.4)	.181^*b*^
Education, y, mean (SD)	3.7 (3.8)	4.4 (2.7)	.731^*b*^
Body mass index, mean (SD)	25.8 (3.3)	26.1 (3.8)	.836^*b*^
MMSE, mean (SD)			
Baseline	22.2 (2.1)	19.7 (3.6)	.181^*b*^
Endpoint	24.8 (2.6)	22.0 (3.9)	.18^*b*^
Difference (endpoint-baseline)	2.7 (2.4)	2.3 (4.8)	.836^*b*^
ADAS-cog score, mean (SD)			
Baseline	10.7 (5.1)	15.2 (8.7)	.445^*b*^
Endpoint	8.0 (3.1)	13.1 (11.3)	.836^*b*^
Difference (endpoint-baseline)	−2.7 (3.3)	−2.2 (6.1)	.628^*b*^

Abbreviations: ADAS-cog score, Alzheimer Disease Assessment Scale-Cognitive Subscale; MMSE, Mini-Mental State Examination.

^a^Fisher exact test.

^*b*^Mann‒Whitney U test, if the distribution was not normal.

### Patients

The detailed methods have been described elsewhere (Lin et al., 2014). Patients were evaluated by research psychiatrists and neurologists after a thorough medical and neurological workup. Inclusion criteria included being aged between 50 and 90 years; probable AD (by National Institute of Neurological and Communicative Diseases and Stroke/Alzheimer’s Disease and Related Disorders Association criteria) (McKhann et al., 1984); Clinical Dementia Rating (Morris, 1993) scores of 1; Mini-Mental State Examination (MMSE) (Folstein et al., 1975) scores of 17 to 26; being physically healthy, with laboratory assessments (including urine/blood routine, biochemical tests) within normal limits; sufficient education to communicate effectively; being capable of completing the assessments of the study; agreeing to participate in the study; and providing informed consent. For patients who had already been on a regimen of antidementia therapy, the therapy had to be continued for at least 3 months before enrollment, and the dose had to be kept unchanged during the study duration. Patients who had not yet been taking antidementia medications were forbidden to do so during the study duration.

Exclusion criteria included history of significant cerebrovascular disease; Hachinski Ischemic Score >4; major neurological, psychiatric, or medical conditions other than AD; substance (including alcohol) abuse or dependence; delusion, hallucination, or delirium symptoms; severe visual or hearing loss; and inability to follow protocol.

### Study Design

All patients were randomly and double-blindly assigned to receive a 24-week treatment of sodium benzoate or placebo in a 1:1 ratio. Efficacy and safety were evaluated at baseline and at the end of weeks 8, 16, and 24. Medication was provided with identical-appearing capsules of sodium benzoate or placebo. The dose was initiated at 250–500 mg/d in the first 8 weeks and escalated by 250–500 mg/d every 8 weeks, if clinically indicated. The primary outcome measure was the Alzheimer Disease Assessment Scale-Cognitive Subscale (ADAS-cog) (Rosen et al., 1984).

### Sample Preparation

Laboratory parameters were measured at baseline and the end point. The albumin and immunoglobulin (IgG) in 25 μL of serum sample were depleted by ProteoPrep Blue Albumin and IgG Depletion kits (PROTBA-1KT, Sigma-Aldrich). The albumin-/IgG-depleted serum sample was purified with cold (−20°C) acetone at −20°C overnight and centrifuged at 12 000× g at 4°C for 10 minutes. After removal of the supernatant, the protein pellet was dissolved in 8 M urea/2 M thiourea solution and quantitated by Bradford assay.

### Protein OFFGEL Fractionation

The purified protein sample (100 μg) from each patient was prepared in a 3.6-mL sample volume and fractionated by a 3100 OFFGEL fractionator (Agilent Technologies, Palo Alto, CA, USA) according to the manufacturer’s instructions. The 24-cm IPG strip (pH 3-10, GE Healthcare, Bio-Sciences) was fixed on a 24-well loading tray. The IPG strip was rehydrated with 40 μL of OFFGEL stock solution for 15 minutes. Then, 150 μL of sample volume was loaded into each well and run for a total power of 64 KVhr using a constant electric current of 50 μA. After focusing, the separated proteins in 24 fractions were purified with cold (−20°C) acetone at −20°C overnight and centrifuged at 12 000× g at 4°C for 10 minutes. The protein pellets were dissolved in a solution containing 8 M urea and 2 M thiourea and quantitated by Bradford assay.

### In-Solution Digestion

The purified proteins (2 μg) from each fractionated sample were dissolved in 20 μL of 25 mM triethylammonium bicarbonate (pH 8.5) and reduced with 2 μL of 50 mM dithiothreitol (DTT) at room temperature for 45 minutes, followed by the addition of 6 μL of 50 mM iodoacetamide (IAA) in the dark at room temperature for 45 minutes. Triethylammonium bicarbonate (25 mM) was further added to the protein solution to reduce the urea concentration to below 1 M. Samples were digested with trypsin (1:10 trypsin to protein ratio in weight) at 37°C overnight. The tryptic peptides were purified by a C18 ZipTip (Millipore) and dried in a centrifugal concentrator. The dried samples were stored at −80°C until nanoLC MS/MS analysis.

### NanoLC‒MS/MS Analysis

The tryptic peptides (500 ng) were analyzed by a nanoflow ultra-performance liquid chromatography system (UltiMate 3000 RSLCnano system; Dionex) coupled to a hybrid quadrupole time-of-flight mass spectrometer (maXis Impact, Bruker). After sample loading, the peptides were eluted from a trap column (Acclaim PepMap C18, 5 μm, 100 Å, 20 μm × 100 mm; Thermo Scientific) into an analytical column (Acclaim PepMap C18, 2 μm, 100 Å, 75 μm × 250 mm; Thermo Scientific) coupled to a nanoelectrospray ionization source (CaptiveSpray) (Lee et al., 2021) on the quadrupole time-of-flight mass spectrometer. The nanoLC gradient conditions were as follows: 10% to 40% (v/v) buffer B (80% acetonitrile (ACN)/0.1% formic acid (FA)) for 68 minutes and then to 99% B for 0.1 minutes; held at 99% B for 5 minutes, then returned to 90% buffer A (2% ACN/0.1% FA) for 10 minutes.

Data-dependent acquisition (DDA) was used to acquire precursor ions and their corresponding fragmented ions. Seven precursors of charge +2, +3, and +4 from each TOF MS scan were dynamically selected and isolated for MS/MS fragment ion scanning. The selected precursors were then actively excluded for 15 seconds. The MS and MS/MS accumulation were set at 2 and 10 Hz, respectively. The cycle time of MS and 7 MS/MS scans was 1.2 seconds.

### Data Analysis

The DDA data files from the nanoLC‒MS/MS system were imported into PEAKS software (PEAKS studio X, Bioinformatics Solutions Inc.) for protein identification by searching against the UniProt database. The search parameters for precursor ion and fragment ion tolerance were 80 ppm and 0.05 Da, respectively. The following search parameters were selected: taxonomy, human; missed cleavages, 2; enzyme, trypsin; fixed modifications, carbamidomethyl (C); and variable modifications, oxidation (M) and deamidation (NQ). The false discovery rate (FDR) was <1%. The label-free function by DDA was performed for protein quantification in PEAKS software to obtain the relative abundance of proteins among groups. The quality score of 8 was used in PEAKS software to select high quality peptides for peptide and protein quantitation. Pearson correlation was performed to analyze the correlation between the abundance of protein candidates and MMSE or ADAS-cog scores across all patients. All statistical analyses were performed using SigmaPlot (version 12.3).

## RESULTS

A total of 13 patients with mild AD were recruited. Among them, 6 patients received sodium benzoate and 7 received placebo. The demographic and clinical characteristics are shown in Table 1. Patients in the sodium benzoate group and the placebo groups did not differ significantly in sex distribution, age, education level, body mass index, anti-dementia medication (donepezil) dose, or ADAS-cog scores at baseline. With the limited sample size, the benzoate group showed a numerical, albeit insignificant, trend of higher MMSE increment (2.7 ± 2.4 vs 2.3 ± 4.8, *P* = .84) and ADAS-cog score reduction (2.7 ± 3.3 vs 2.2 ± 6.1, *P* = .63).

### Serum Proteomics by OFFGEL Fractionation and Label-Free Quantification

**Figure 1.**
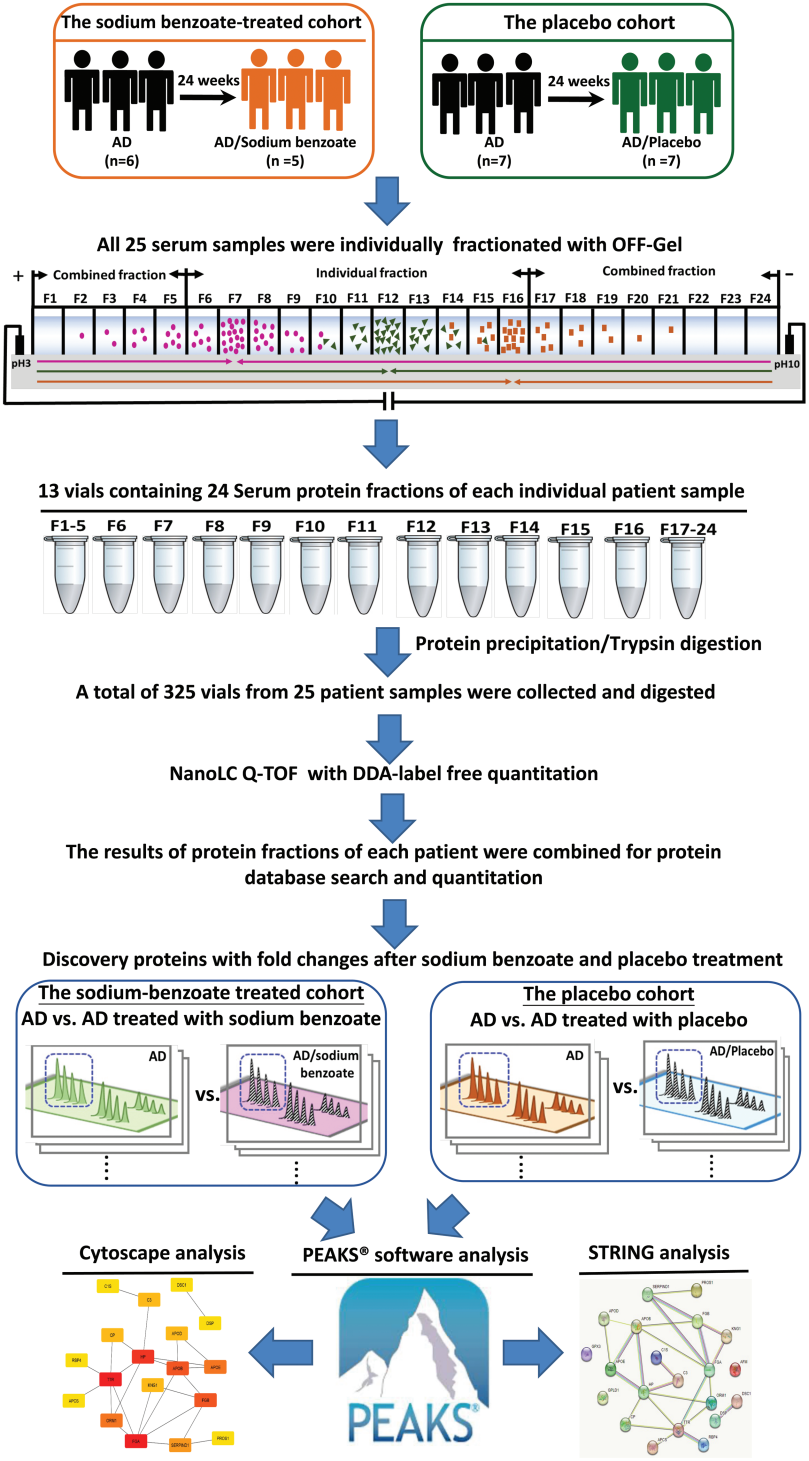
Flowchart of using off-gel fractionation and label-free quantitative proteomics to analyze serum samples from the cohort study of the AD patient-sodium benzoate-treated group and the placebo group.

**Figure 2.**
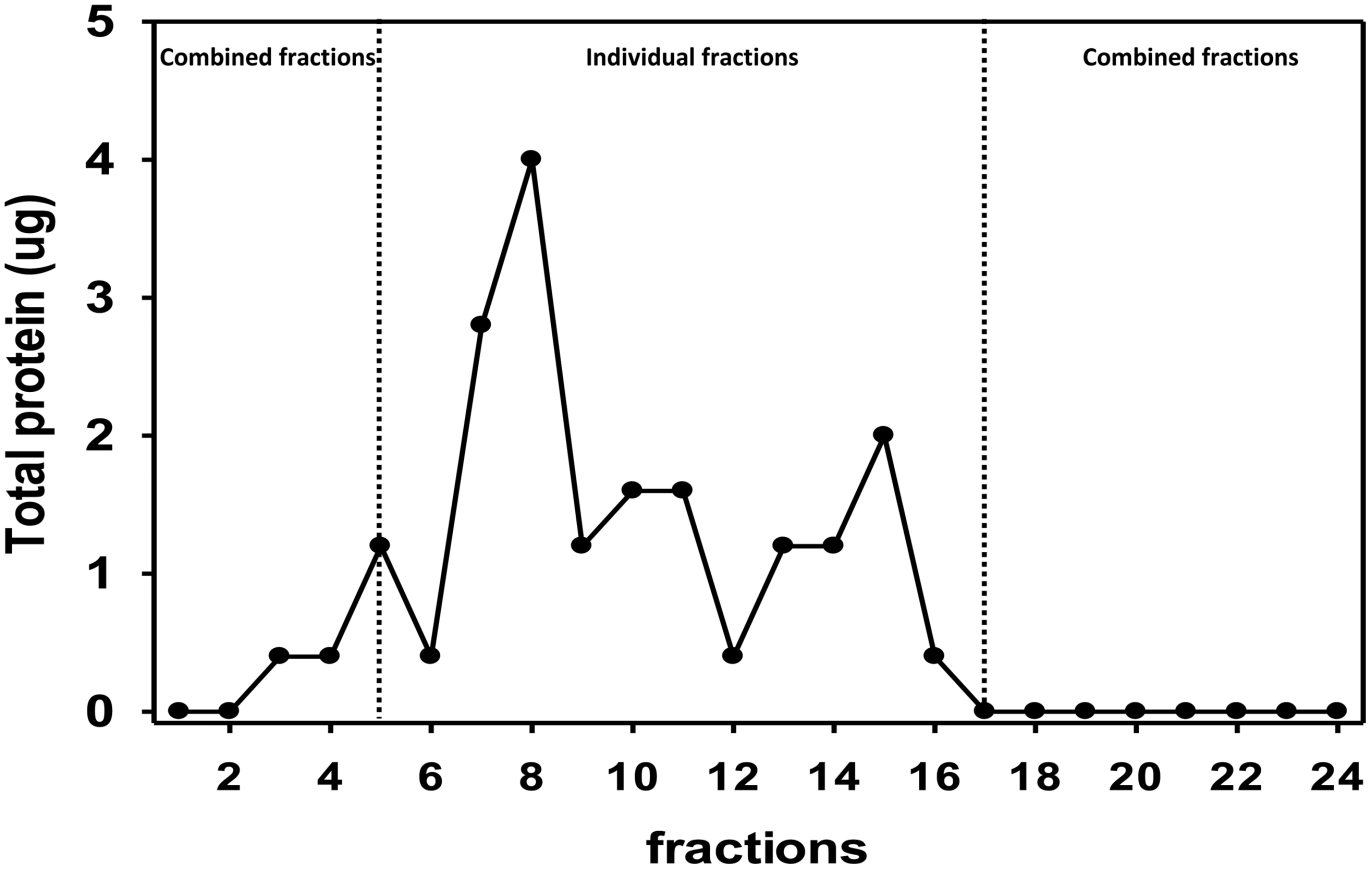
The serum protein content in fractions 1 to 24 separated by OFFGEL fractionation.

To discover protein biomarkers that exhibited changes between patients treated with benzoate and those with a placebo, serum protein samples were obtained from each patient and purified. The purification process involved fractionation using an OFFGEL fractionator, which is based on IEF separation. The fractions were then subjected to protein digestion and label-free nanoLC‒MS proteomics with the DDA function. The workflow for this process is depicted in Figure 1. As shown in Figure 2, abundant proteins were mainly located in fractions 6–16. Therefore, fractions 1–5 were pooled together as a single sample, fractions 17–24 were pooled as another sample, and fractions 6–16 were collected as individual samples without pooling. These samples were then subjected to protein digestion and nanoLC‒MS/MS analysis.

### Proteins With Fold Changes in Follow-Up AD Cohorts of Sodium Benzoate-Treated and Placebo Groups

**Figure 3.**
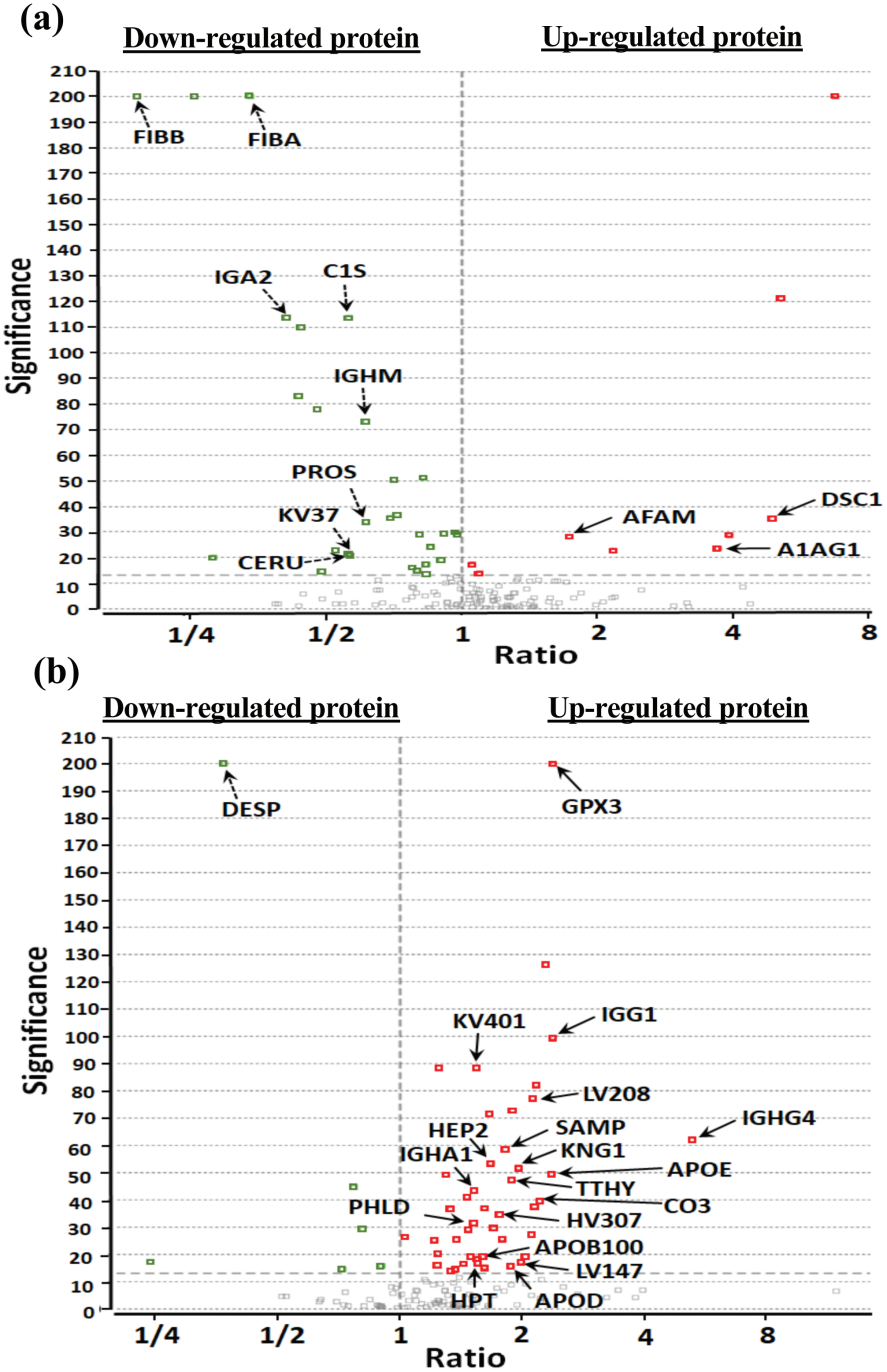
Volcano plot of proteins with expressed fold changes. (A) sodium benzoate-treated cohort. (B) Placebo cohort. Ratios were expressed as (24 weeks treatment)/(baseline).

A total of 861 proteins were identified in the benzoate treatment group, while 927 proteins were identified in the placebo groups (supplementary Table 1). To identify proteins with significant expression changes in the cohort of the benzoate group and the placebo group, a volcano plot was generated (Figure 3). Three proteins (Desmocollin-1 [DSC1], Afamin [AFM or AFAM], and Alpha-1-acid glycoprotein 1 [ORM1 or A1AG1]) were found to be upregulated with a ratio ≥1.5 after benzoate treatment. (Figure 3A) (supplementary Table 2). However, these 3 proteins did not exhibit significant fold changes in the placebo group. Eight proteins (Immunoglobulin heavy constant mu [IGHM], Fibrinogen beta chain [FGB or FIBB], Complement C1s subcomponent [C1S], Probable non-functional immunoglobulin kappa variable 3-7 [KV37], Ceruloplasmin [CP], Immunoglobulin alpha-2 heavy chain [IGA2], Vitamin K-dependent protein S [PROS1], and Fibrinogen alpha chain [FGA or FIBA]) were found to be downregulated with a ratio ≤0.67 after benzoate treatment. Among these, 6 proteins (IGHM, FIBB, C1S, KV37, CERU, and IGA2) did not show significant fold changes in the placebo treatment group. Two proteins (PROS1 and FIBA) that were upregulated with a ratio ≥1.5 were found to be elevated in the placebo group (supplementary Table 2). Additionally, 1 protein (Retinol-binding protein 4 [RBP4 or RET4]) was upregulated with a ratio ≥1.5 in the placebo group; however, it was downregulated with a ratio ≤0.67 after benzoate treatment. Nineteen proteins were found to be upregulated with a ratio ≥1.5 in the placebo group. (Figure 3B) Among them, only RET4 was found to be downregulated, but the other 18 proteins did not show significant fold changes after benzoate treatment. Another protein, Desmoplakin (DSP), was found to be downregulated with a ratio ≤0.67 in the placebo group, but this protein did not show significant fold changes after benzoate treatment.

### Identification of Key Protein Networks and Processes Associated With Differential Protein Expression

**Figure 4.**
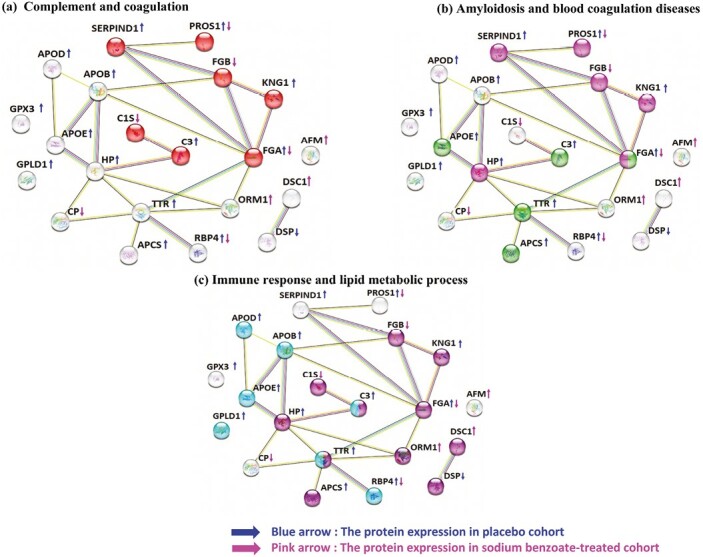
STRING analysis of proteins with significant fold changes. Proteins with fold changes in the sodium benzoate-treated group and placebo group are presented using red and blue arrows, respectively. (A) The proteins involved in complement and coagulation cascades are marked in red. (B) The proteins involved in amyloidosis and blood coagulation diseases are marked in green and purple, respectively. (c) The proteins involved in the immune response and lipid metabolic process are marked in purple and blue, respectively.

**Figure 5.**
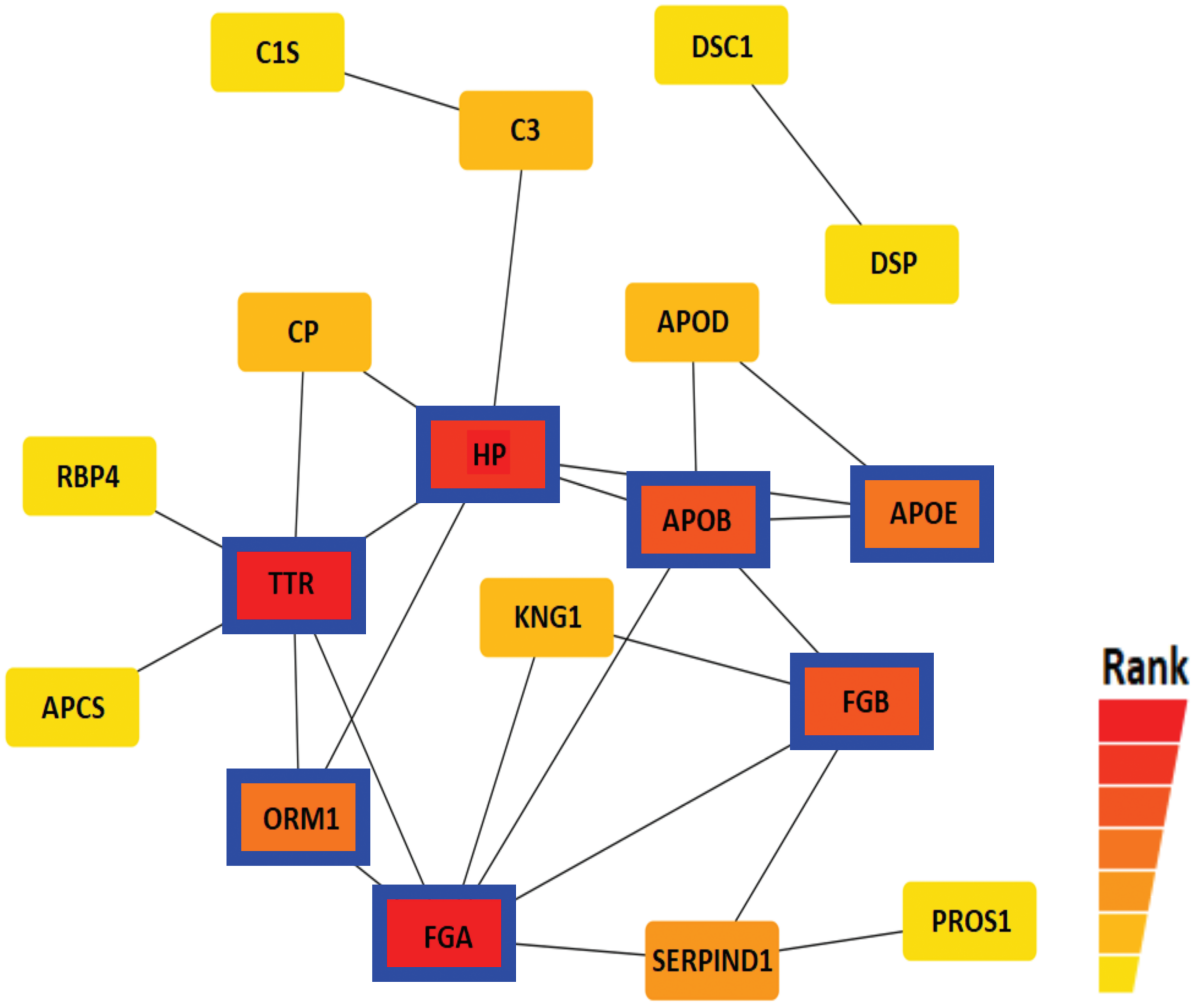
Cytoscape analysis of the proteins with significant fold changes.

To identify the most influential and potential interactions among proteins with significant fold changes, all proteins with differential changes (including both up- and downregulated) were analyzed using STRING. Several enriched clusters emerged from this analysis. Notably, the proteins associated with the complement and coagulation cascades (PROS1, Heparin cofactor 2 [SERPIND1], FGB, Kininogen-1 [KNG1], FGA, C1S, and Complement C3 [C3]) were significantly enriched in the Gene Ontology (GO) biological process and KEGG pathways (FDR = 1.17E-9) (Figure 4A). Furthermore, the disease-gene associations related to amyloidosis (Apolipoprotein E [APOE], C3, FGA, Transthyretin [TTR], and Serum amyloid P-component [APCS or SAMP]) showed significant enrichment (FDR = 2.66E-5) (Figure 4B). In addition, we observed enrichment of proteins involved in immune responses (FGB, KNG1, FGA, ORM1, TTR, APCS, C3, Haptoglobin (HP), C1S, DSC1, and DSP) and lipid metabolic processes (Apolipoprotein B-100 [APOB], APOD, APOE, C3, TTR, Phosphatidylinositol-glycan-specific phospholipase D [GPLD1], and RBP4) (FDR = 9.91E-5 and FDR = 0.0176, respectively) (Figure 4C). To further explore the functional enrichment networks, we conducted an analysis using Cytoscape, which revealed that TTR, FGA, HP, APOB, FGB, APOE, and ORM1 acted as hub proteins within the protein‒protein interaction networks (Figure 5). These findings highlight the central role of these proteins and their interactions in the context of the studied processes.

Pearson correlation between the abundance of 31 protein candidates and MMSE or ADAS-cog scores was evaluated. ORM1 showed a significant positive correlation with MMSE scores (*r* = 0.48, *P* = .02), while the other 30 protein candidates had no significant correlations with MMSE scores (with *r* values ranging from −0.22 to 0.32) (supplementary Figure 1).

DSP (*r* = −0.42, *P* = .04), FGA (*r* = −0.4, *P* = .05), and HP (*r* = 0.45, *P* = .02) were correlated with ADAS-cog scores, whereas the other 28 candidates had no significant correlations with ADAS-cog scores (r values ranging from −0.32 to 0.34) (supplementary Figure 2).

## DISCUSSION

In this study, we analyzed and compared the serum proteomes of participants treated with either benzoate or a placebo. We focused on proteins that showed elevated levels in the placebo group but decreased levels in the benzoate group or had no significant fold changes. These proteins can provide insights into the potential mechanisms of action of sodium benzoate. Our proteomic results revealed that sodium benzoate had a profound impact on the complement and coagulation cascade pathways, disease-gene associations related to amyloidosis, and biological processes of immune responses and lipid metabolism.

### Complement, Immune, and Coagulation Cascades

Our findings indicated that PROS1, SERPIND1, KNG1, FGA, and C3 were elevated in the placebo group, whereas PROS1, FGB, FGA, and C1S proteins were decreased in the benzoate group. SERPIND1, KNG1, and C3 proteins showed no significant fold changes after 24 weeks of benzoate treatment. This suggests that the placebo and benzoate treatments have distinct effects on the complement and coagulation systems. The complement system is an integral part of the innate immune response and plays a role in the clearance of Aβ plaques in the brain. Previous studies have shown elevated levels of complement proteins, including C1q, C3, and C4, in AD brain tissue (Shen et al., 2001; Fonseca et al., 2011). However, the role of complement in AD is controversial, as it can also contribute to neuroinflammation and neuronal damage (Ricklin et al., 2010; Tenner et al., 2018). The increased levels of complement proteins in the placebo group might reflect an ongoing immune response to Aβ plaques in the brain. Therefore, in the placebo group, HP, TTR, RBP4, and APCS proteins associated with immune responses were also found to be elevated, except for C3. However, the benzoate-treated group showed decreased levels of C1S and RBP4 proteins, whereas C3, HP, TTR, and APCS proteins had no significant fold changes. The elevation of these complement proteins in the placebo group may reflect an ongoing immune response to Aβ plaques in the brain.

Stress and inflammation can activate the coagulation and complement systems, leading to an increase in the levels of coagulation and complement proteins (Mackman, 2004; Klos et al., 2009). The decrease in PROS1 and FGB/FGA/C1S proteins in the benzoate group could be attributed to the anti-inflammatory properties of benzoate, which might have dampened the inflammatory response that typically leads to an increase in these proteins (Brahmachari et al., 2009; Oshima et al., 2023).

Haptoglobin (HP) has been identified as an indicator of blood‒brain barrier (BBB) integrity, providing valuable insights into its disruption in AD (Chamoun et al., 2001). Additionally, HP plays a crucial role in facilitating the formation of the stable complex between APOE and Aβ, 2 key proteins implicated in AD. This finding highlights HP as a potential contributor to the development and progression of AD (Spagnuolo et al., 2014). Clinical studies have reported higher levels of HP in the cerebrospinal fluid (CSF) (Johnson et al., 1992) and serum (Zhu et al., 2018) of patients with AD than in healthy controls.

### Amyloidosis

In the placebo group, TTR, APCS, FGA, C3, and APOE proteins were increased after 24 weeks of placebo treatment. However, FGA was decreased, and TTR, APCS, C3, and APOE showed no significant fold changes in the benzoate-treated group. Amyloidosis is characterized by protein misfolding leading to amyloid plaques and has a strong association with the serum amyloid P component (APCS or SAP). SAP, a glycoprotein, is known to bind Aβ peptides and stabilize them against proteolysis (Mold et al., 2012). Increased plasma concentrations of SAP have been linked to impaired cognitive performance (Nybo et al., 1998). Elevated brain SAP concentrations may contribute to the development of neurodegenerative diseases due to damaged BBR integrity (Chen et al., 2012).

TTR, a tetrameric protein, is mainly synthesized in the liver and the choroid plexus of the brain. It circulates at varying concentrations in both plasma and CSF. TTR serves as the major binding protein for Aβ in CSF and plays a crucial role in breaking down Aβ aggregates, thus inhibiting the formation of Aβ plaques (Chen et al., 2012). The TTR-Aβ (1–42) complex is released across the BBB through low-density lipoprotein receptor-related protein 1 (LRP1), which is 1 of the receptors involved in controlling substrate efflux at the BBB. Consequently, TTR is believed to have a protective role in AD. The elevated levels of plasma TTR in the placebo group may reflect pathological alterations in the brain due to a leaky BBB (Sweeney et al., 2018).

Fibrinogen is an inactive precursor of fibrin, the primary protein component of blood clots. Damage to the BBB allows fibrinogen to enter the brain parenchyma and accumulate, contributing to AD development (Cortes-Canteli and Strickland, 2009). Fibrinogen can interact with Aβ, leading to fibrinogen oligomerization, increased deposition of fibrinogen, and enhanced Aβ fibrillization (Ahn et al., 2010). Structural changes induced by Aβ in fibrin clots result in increased resistance to enzymatic degradation and thrombosis and prolonged persistence of fibrin in the brain (Cortes-Canteli et al., 2010; Zamolodchikov and Strickland, 2012).

APOE is a lipid transporter in the plasma and the central nervous system. It exists in 3 common allelic isoforms: APOE2, APOE3, and APOE4. APOE4 is a major risk factor for AD. Intensive research has uncovered potential mechanisms by which APOE isoforms contribute to AD development, including Aβ binding and plaque formation, induction of membrane disruption, and increased neuronal sensitivity to injury (Hatters et al., 2006). Plasma APOE levels have been reported to be higher in the AD group than in healthy controls (Laffoon et al., 2022).

### Lipid Metabolic Process

APOB, APOD, APOE, C3, TTR, GPLD1, and RBP4, all involved in lipid metabolic processes, showed elevated levels in the placebo group and exhibited no significant fold changes in the benzoate-treated group. Abnormal accumulation of apolipoproteins and cholesterol has been detected as core components of mature amyloid plaques in the brains of AD patients (Bereczki et al., 2008). Apolipoprotein B100, a major component of chylomicrons and low-density lipoprotein, has been reported to be elevated in the serum of AD patients (Caramelli et al., 1999; Sabbagh et al., 2004). Overexpression of human APOB-100 in transgenic mice increases serum lipid levels, leading to cerebrovascular lesions and subsequent apoptosis and neurodegeneration (Bereczki et al., 2008).

This study had several limitations. First, a small sample size was used in this study, because a huge data size produced from subfractions of each sample can crash the quantification process in the PEAKS software. Second, with the limited sample size, the benzoate group showed a numerical, albeit insignificant, trend of higher MMSE increment and ADAS-cog score reduction. Third, whether the blood levels of the proteins can reflect their levels in brain deserves study. Fourth, further studies are needed to decider the underlying mechanisms of the effects of these proteins on cognitive functions of the patients with AD. Finally, we recruited only Han Taiwanese patients in this study. More studies are required to examine whether ethnic difference may influence the results.

## CONCLUSIONS

As an NMDAR enhancer, sodium benzoate has been reported to improve the cognitive function of patients with AD in randomized, double-blind, placebo-controlled trials (Lin et al., 2014; Lane et al., 2023). The study explored the potential mechanisms of sodium benzoate in treating AD using label-free quantitative proteomics with a small sample size. The results indicated that sodium benzoate affects important pathways associated with AD, including complement and coagulation cascades, amyloidosis disease, immune responses, and lipid metabolic processes. The hub proteins identified in the protein‒protein interaction networks included TTR, FGA, HP, APOB, FGB, APOE, and ORM1. These findings suggest that sodium benzoate may have a significant impact on the pathogenesis of AD. The study also highlighted the use of proteomics for analyzing serum samples in AD patients, providing insights into disease biomarkers, potential therapeutic targets, and disease mechanisms. By fractionating the serum proteins and subjecting them to nanoLC‒MS/MS analysis, the study achieved more comprehensive proteome profiling. Overall, sodium benzoate shows promise as a novel, safe, and efficient therapy for AD patients; the proteins with significant fold changes in the sodium benzoate-treatment group compared with the placebo group could also be used to monitor the progression of treatment efficacy.

## Supplementary Material

pyad061_suppl_Supplementary_Figure_S1

pyad061_suppl_Supplementary_Figure_S2

pyad061_suppl_Supplementary_Table_S1

pyad061_suppl_Supplementary_Table_S2

## Data and Materials Availability

The datasets supporting the conclusions of this article are available in the [MassIVE] repository, **[**MSV000092141, https://massive.ucsd.edu/ProteoSAFe/static/massive.jsp].
