# Erratum to: Elucidating the Mechanisms of Sodium Benzoate in Alzheimer Disease: Insights from Quantitative Proteomics Analysis of Serum Samples

**DOI:** 10.1093/ijnp/pyae030

**Published:** 2024-07-15

This is an erratum to: Chieh-Hsin Lin, Hsin-Yi Liao, Hsien-Yuan Lane, Chao-Jung Chen, Elucidating the Mechanisms of Sodium Benzoate in Alzheimer Disease: Insights from Quantitative Proteomics Analysis of Serum Samples, *International Journal of Neuropsychopharmacology*, Volume 26, Issue 12, December 2023, Pages 856–866, https://doi.org/10.1093/ijnp/pyad061

The corresponding author of this paper has been changed from Hsien-Yuan Lane to Chao-Jung Chen.

