# Supplementary material for: Elucidating the Mechanisms of Sodium Benzoate in Alzheimer Disease: Insights from Quantitative Proteomics Analysis of Serum Samples
Source: Int J Neuropsychopharmacol. 2023 Oct 24;26(12):856–66. doi: 10.1093/ijnp/pyad061 (PMC10726399; doi:10.1093/ijnp/pyad061)
Supplement: pyad061_suppl_Supplementary_Figure_S1 [file pyad061_suppl_supplementary_figure_s1.pptx]

## Slide 1
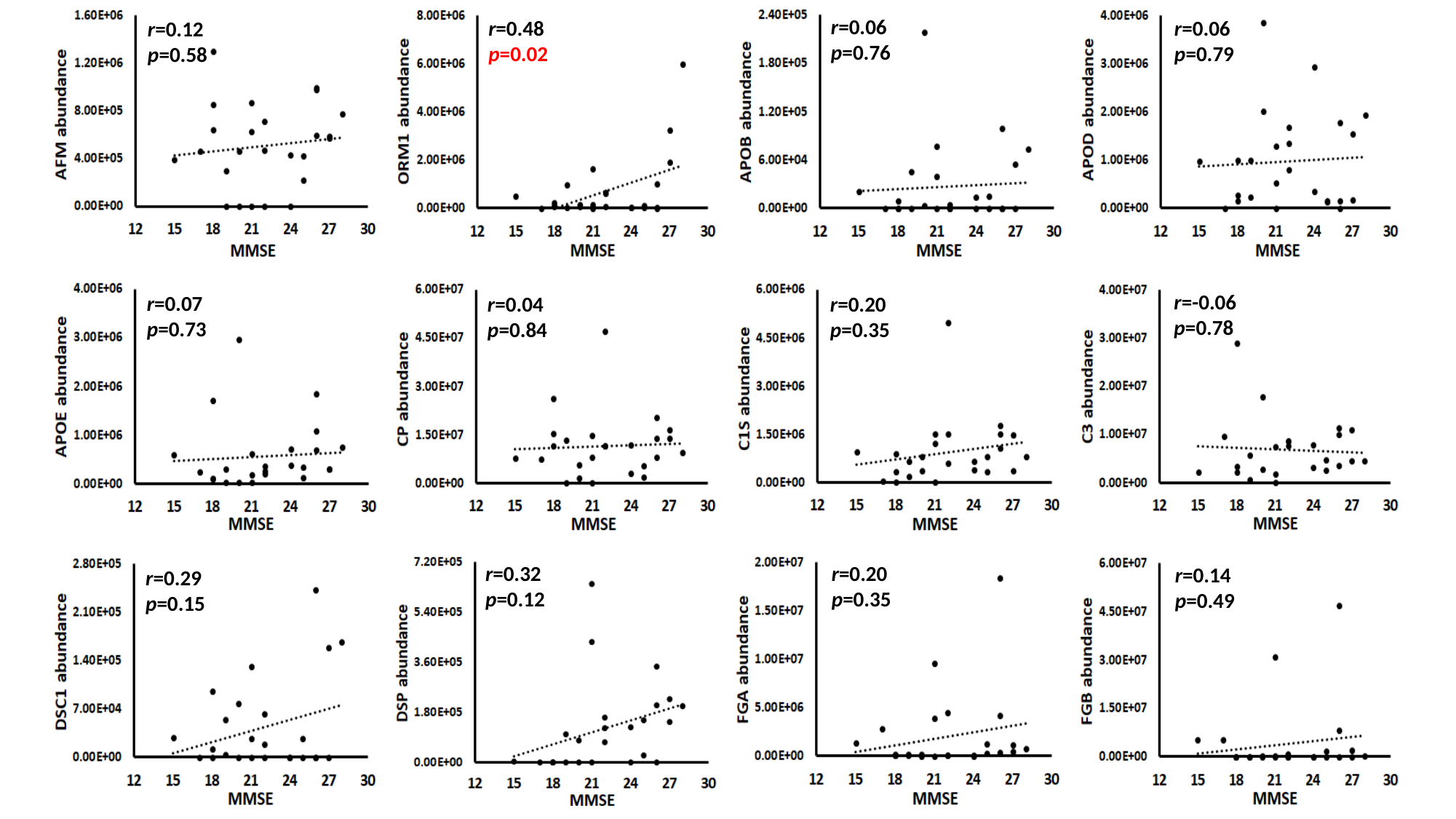

r=0.06
p=0.76
r=0.48
p=0.02
r=0.06
p=0.79
r=0.12
p=0.58
r=-0.06
p=0.78
r=0.07
p=0.73
r=0.04
p=0.84
r=0.20
p=0.35
r=0.20
p=0.35
r=0.32
p=0.12
r=0.14
p=0.49
r=0.29
p=0.15

## Slide 2
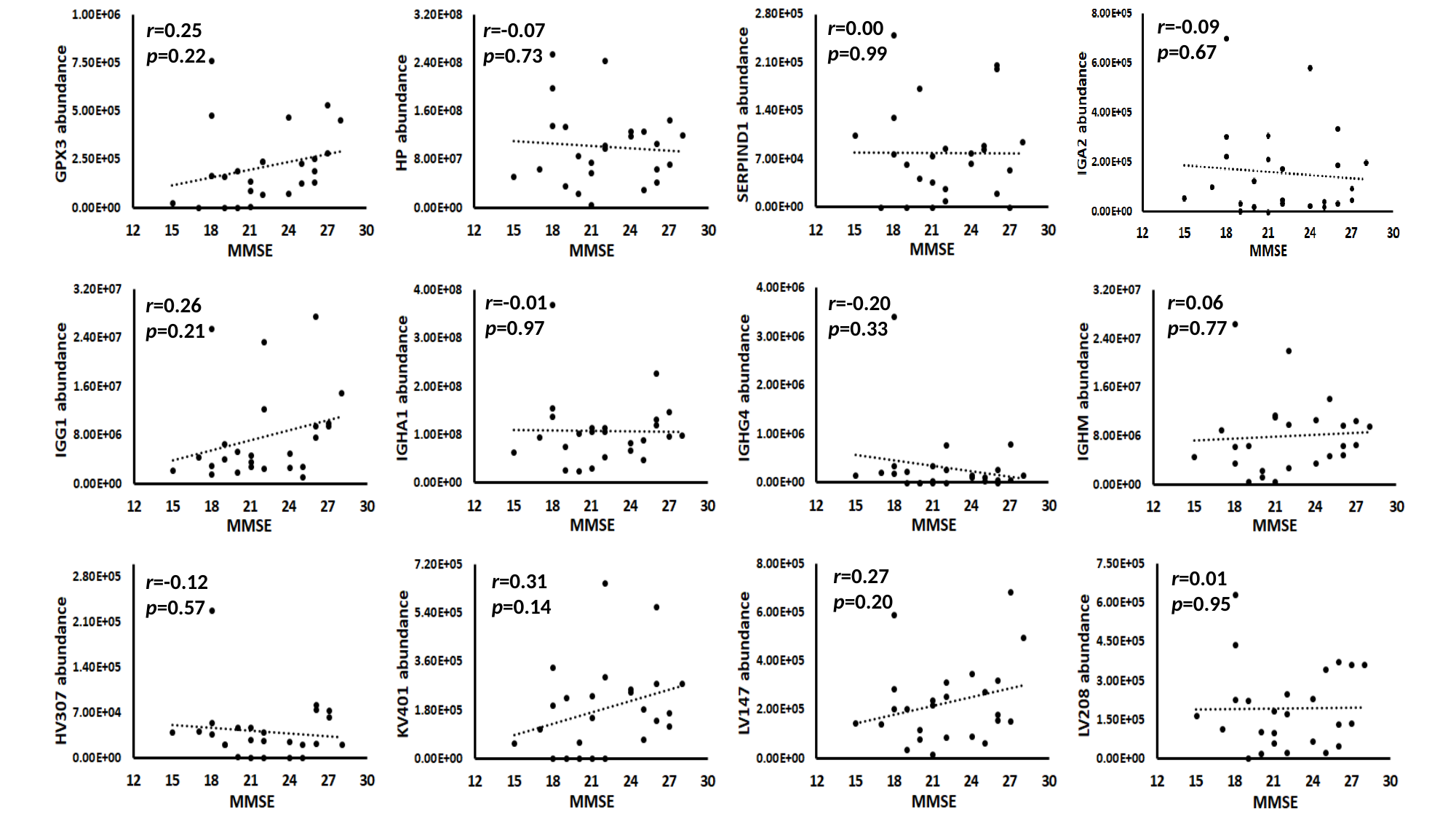

r=-0.09
p=0.67
r=0.00
p=0.99
r=-0.07
p=0.73
r=0.25
p=0.22
r=0.06
p=0.77
r=-0.01
p=0.97
r=-0.20
p=0.33
r=0.26
p=0.21
r=0.27
p=0.20
r=0.01
p=0.95
r=0.31
p=0.14
r=-0.12
p=0.57

## Slide 3
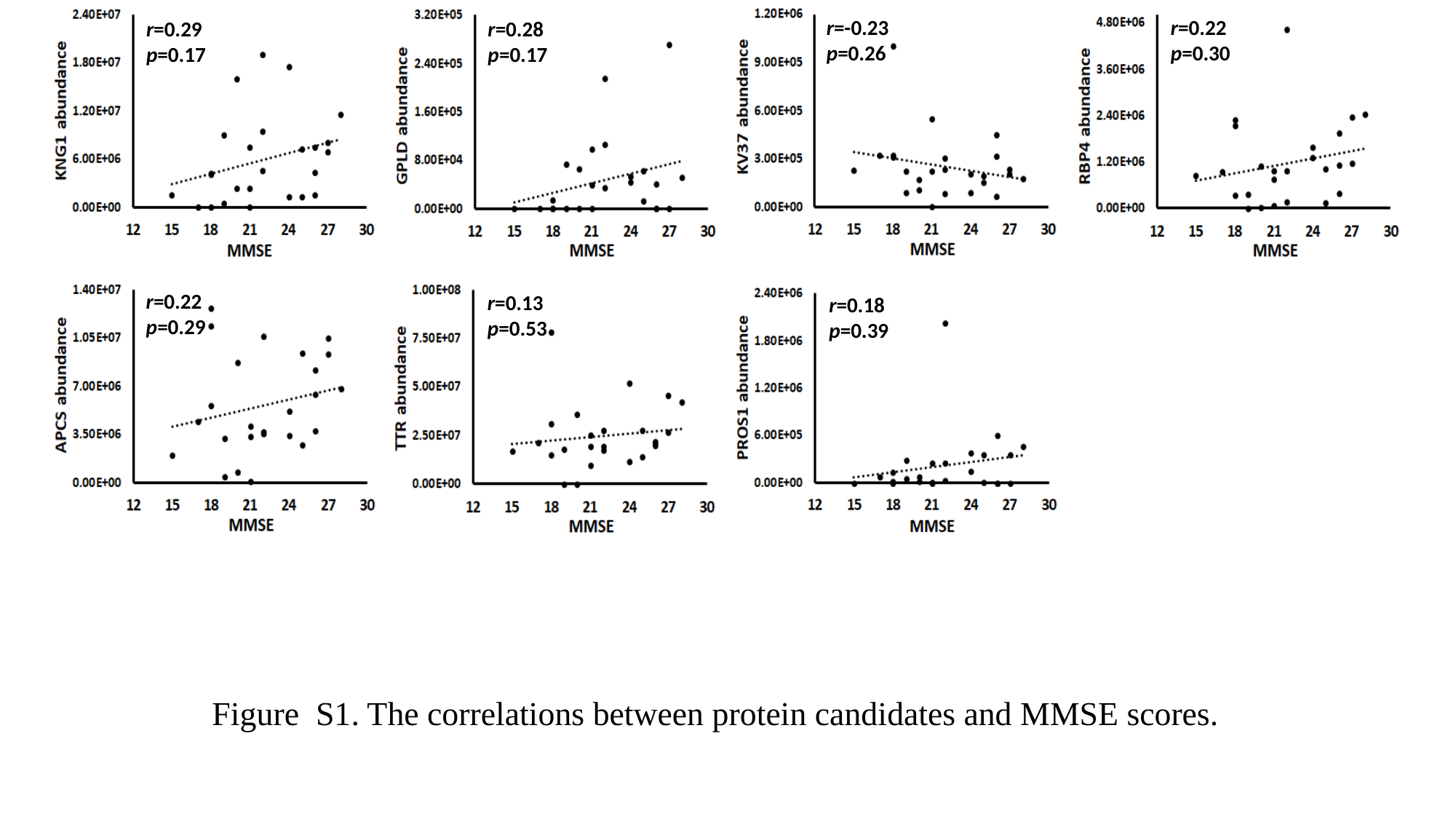

r=-0.23
p=0.26
r=0.22
p=0.30
r=0.28
p=0.17
r=0.29
p=0.17
r=0.22
p=0.29
r=0.13
p=0.53
r=0.18
p=0.39
Figure S1. The correlations between protein candidates and MMSE scores.
