# Supplementary material for: Elucidating the Mechanisms of Sodium Benzoate in Alzheimer Disease: Insights from Quantitative Proteomics Analysis of Serum Samples
Source: Int J Neuropsychopharmacol. 2023 Oct 24;26(12):856–66. doi: 10.1093/ijnp/pyad061 (PMC10726399; doi:10.1093/ijnp/pyad061)
Supplement: pyad061_suppl_Supplementary_Figure_S2 [file pyad061_suppl_supplementary_figure_s2.pptx]

## Slide 1
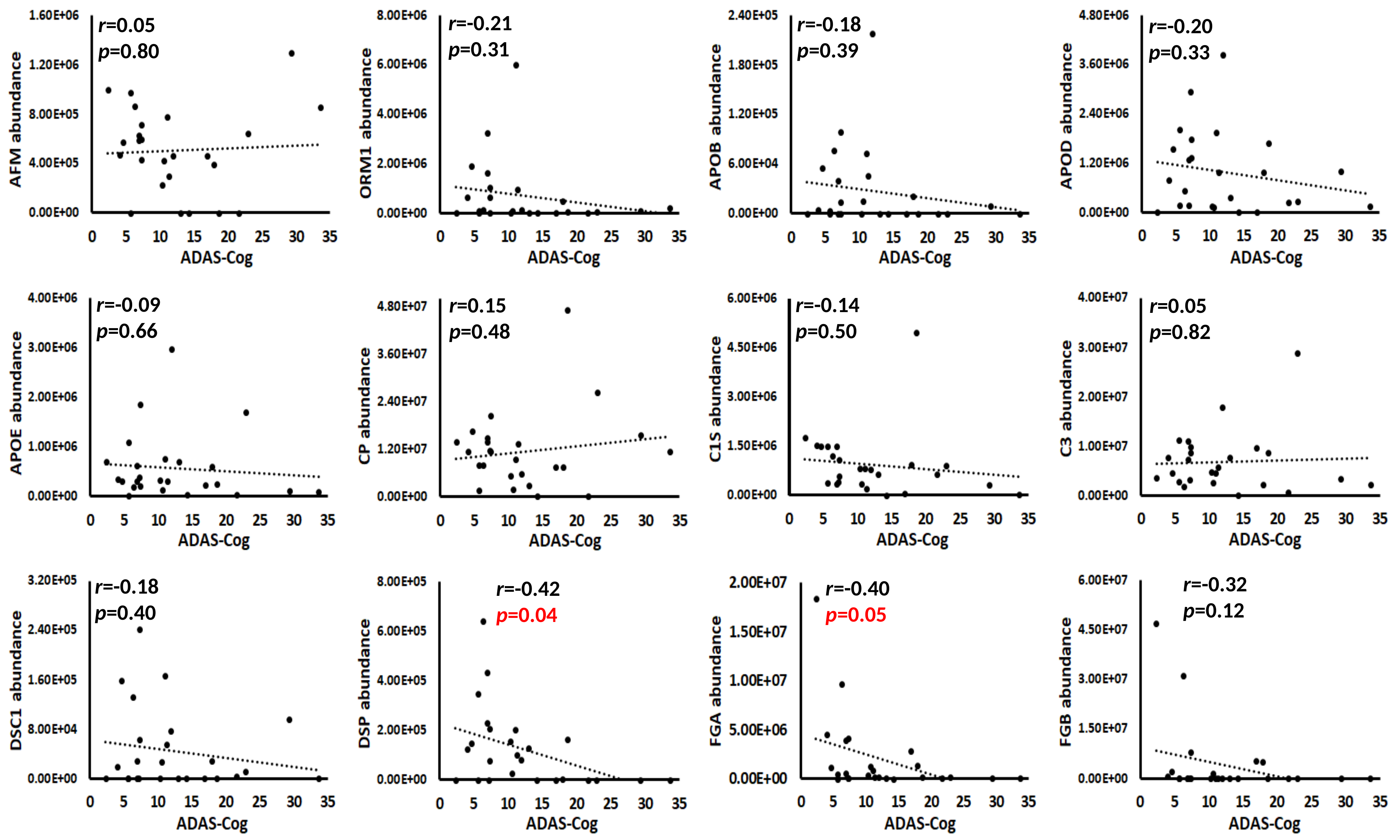

r=-0.21
p=0.31
r=-0.18
p=0.39
r=0.05
p=0.80
r=-0.20
p=0.33
#
r=-0.09
p=0.66
r=-0.14
p=0.50
r=0.15
p=0.48
r=0.05
p=0.82
r=-0.32
p=0.12
r=-0.18
p=0.40
r=-0.40
p=0.05
r=-0.42
p=0.04

## Slide 2
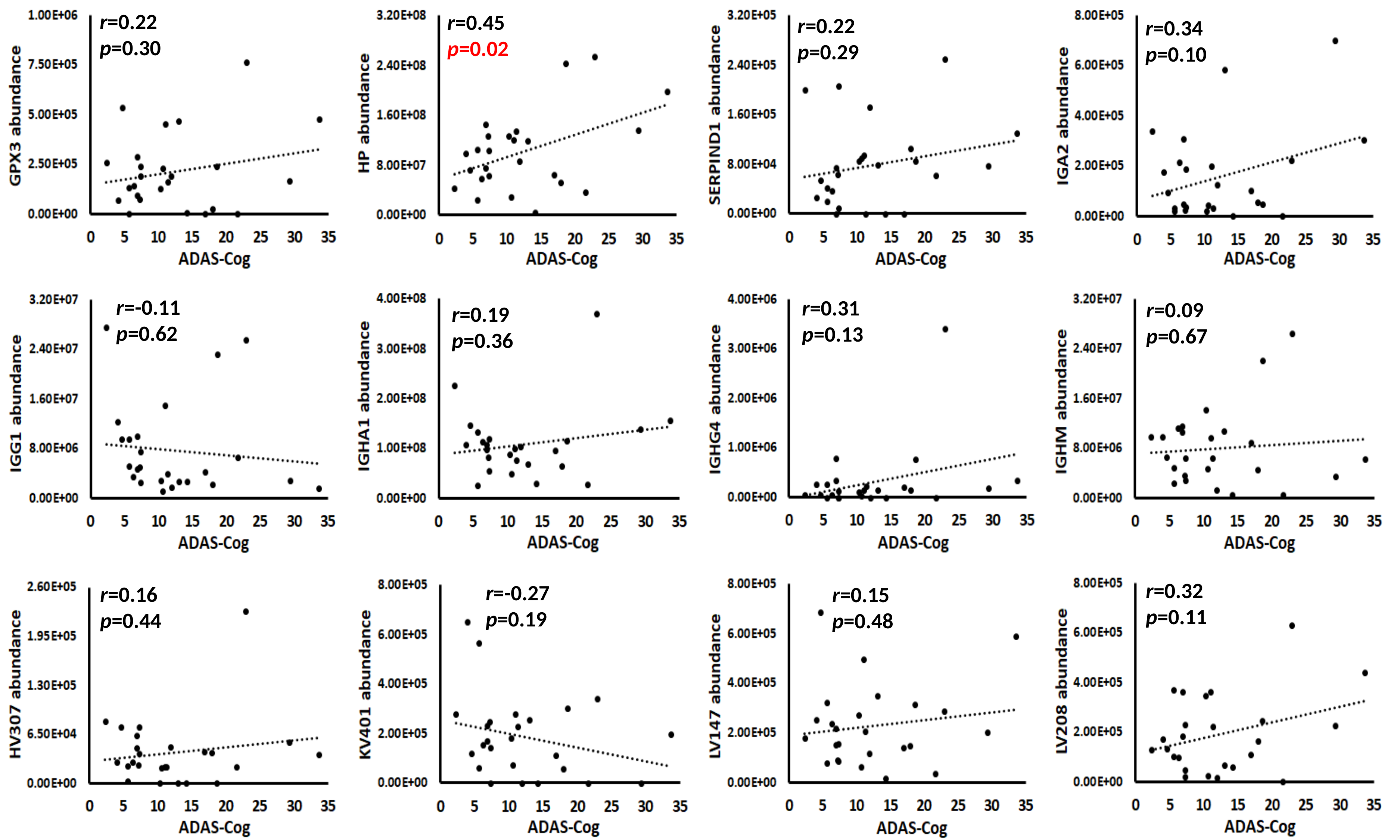

r=0.22
p=0.30
r=0.45
p=0.02
r=0.22
p=0.29
r=0.34
p=0.10
r=-0.11
p=0.62
r=0.31
p=0.13
r=0.09
p=0.67
r=0.19
p=0.36
r=0.32
p=0.11
r=-0.27
p=0.19
r=0.16
p=0.44
r=0.15
p=0.48

## Slide 3
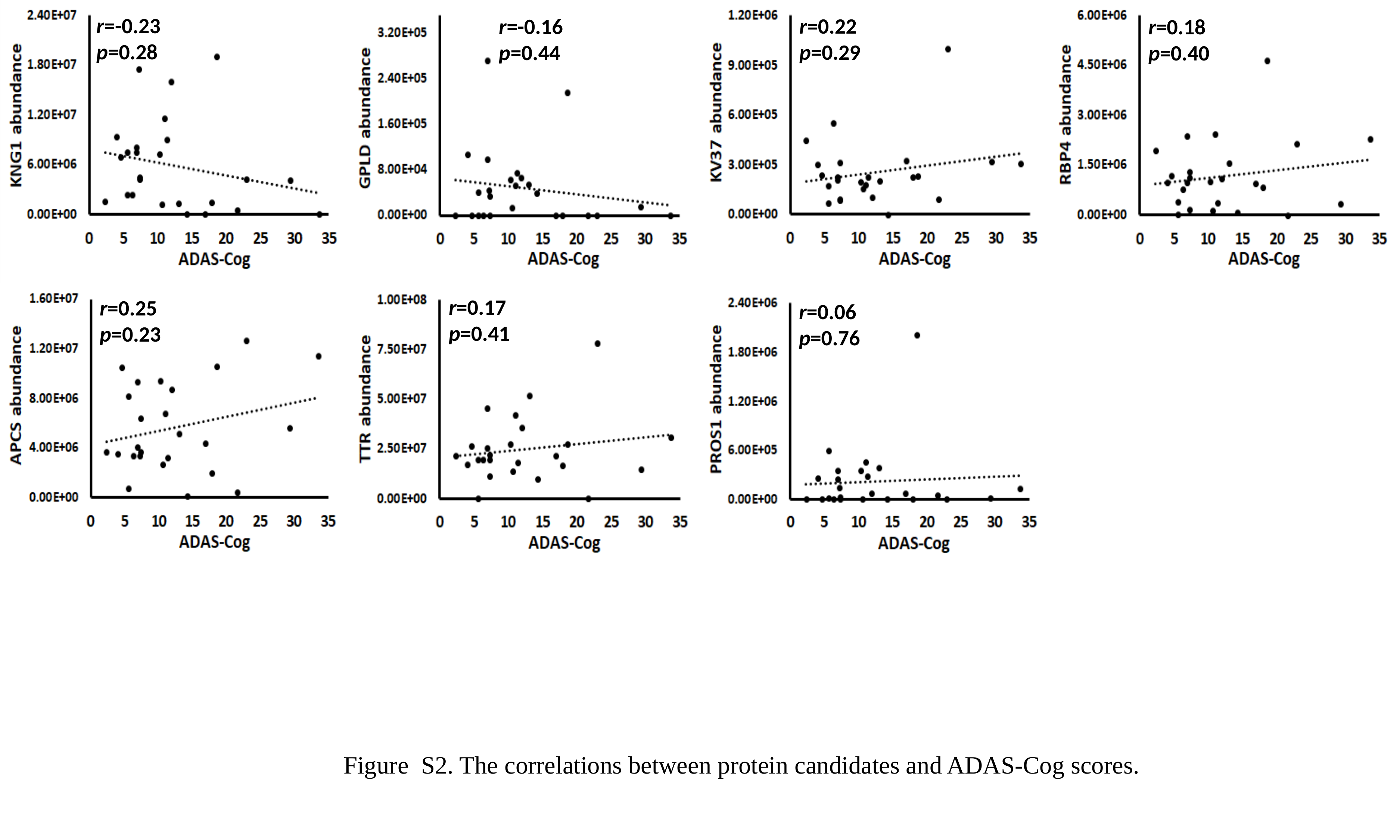

r=-0.23
p=0.28
r=0.22
p=0.29
r=-0.16
p=0.44
r=0.18
p=0.40
r=0.17
p=0.41
r=0.25
p=0.23
r=0.06
p=0.76
Figure S2. The correlations between protein candidates and ADAS-Cog scores.
