# Supplementary material for: Elucidating the Mechanisms of Sodium Benzoate in Alzheimer Disease: Insights from Quantitative Proteomics Analysis of Serum Samples
Source: Int J Neuropsychopharmacol. 2023 Oct 24;26(12):856–66. doi: 10.1093/ijnp/pyad061 (PMC10726399; doi:10.1093/ijnp/pyad061)
Supplement: pyad061_suppl_Supplementary_Table_S2 [file pyad061_suppl_supplementary_table_s2.docx]

**Protein table S2.** List of proteins with significant expression changes in the cohort of the benzoate group and the placebo group.

| **Accession** | **Protein name** | **Avg. Mass** | **Sodium benzoate-treated cohort** | | | **Placebo cohort** | | | |  |
| --- | --- | --- | --- | --- | --- | --- | --- | --- | --- | --- |
|  |  |  | **Significance** | **#Unique** | **Protein ratio**  **(24 weeks treatment/baseline)** | **Significance** | **#Unique** | | **Protein ratio**  **(24 weeks treatment/baseline)** | |
| ***Up-regulated proteins in benzoate-treated cohort*** | | | | | | | | | |  |
| Q08554\|DSC1 | Desmocollin-1 | 99987 | 35.1 | 1 | 4.9 | 15.58 | 2 | 0.9 | |  |
| P43652\|AFAM | Afamin | 69069 | 28.12 | 7 | 1.74 | 0.58 | 6 | 0.91 | |  |
| P02763\|A1AG1 | Alpha-1-acid glycoprotein 1 | 23512 | 23.7 | 3 | 3.7 | 0.59 | 3 | 1.36 | |  |
| ***Down-regulated proteins in benzoate-treated cohort*** | | | | | | | | | |  |
| P01871\|IGHM | Immunoglobulin heavy constant mu | 49440 | 73.23 | 6 | 0.61 | 25.43 | 4 | 1.38 | |  |
| P02675\|FIBB | Fibrinogen beta chain | 55928 | 200 | 2 | 0.19 | 0.65 | 14 | 1.37 | |  |
| P09871\|C1S | Complement C1s subcomponent | 76685 | 113.76 | 11 | 0.56 | 1.35 | 7 | 1.20 | |  |
| A0A075B6H7\|KV37 | Probable non-functional immunoglobulin kappa variable 3-7 | 12783 | 21.6 | 1 | 0.56 | 10.76 | 1 | 1.16 | |  |
| P00450\|CERU | Ceruloplasmin | 122205 | 20.95 | 13 | 0.57 | 2.82 | 15 | 1.06 | |  |
| P0DOX2\|IGA2 | Immunoglobulin alpha-2 heavy chain | 48934 | 113.94 | 1 | 0.41 | 0.97 | 1 | 0.89 | |  |
| P07225\|PROS | Vitamin K-dependent protein S | 75123 | 33.83 | 6 | 0.61 | 2.39 | 1 | 1.56 | |  |
| P02671\|FIBA | Fibrinogen alpha chain | 94973 | 200 | 6 | 0.34 | 0.49 | 7 | 1.52 | |  |
| ***Up-regulated proteins in placebo cohort*** | | | | | | | | | |  |
| P02753\|RET4 | Retinol-binding protein 4 | 23010 | 1.85 | 5 | 0.38 | 71.39 | 3 | 1.67 | |  |
| P04114\|APOB | Apolipoprotein B-100 | 515611 | 0.27 | 1 | 1.44 | 19.29 | 2 | 1.6 | |  |
| P05090\|APOD | Apolipoprotein D | 21276 | 3.08 | 4 | 1.18 | 15.7 | 2 | 1.88 | |  |
| P02649\|APOE | Apolipoprotein E | 36154 | 6.83 | 8 | 1.15 | 49.77 | 7 | 2.37 | |  |
| P01024\|CO3 | Complement C3 | 187147 | 5.09 | 40 | 1.07 | 39.53 | 30 | 2.22 | |  |
| P22352\|GPX3 | Glutathione peroxidase 3 | 25552 | 0.26 | 2 | 0.95 | 200 | 3 | 2.39 | |  |
| P00738\|HPT | Haptoglobin | 45205 | 13.67 | 13 | 0.83 | 16.79 | 10 | 1.57 | |  |
| P05546\|HEP2 | Heparin cofactor 2 | 57071 | 23.85 | 1 | 0.85 | 53.39 | 1 | 1.68 | |  |
| P0DOX5\|IGG1 | Immunoglobulin gamma-1 heavy chain | 49329 | 0.44 | 3 | 1.14 | 99.51 | 3 | 2.40 | |  |
| P01876\|IGHA1 | Immunoglobulin heavy constant alpha 1 | 37655 | 1.55 | 7 | 0.97 | 43.53 | 6 | 1.53 | |  |
| P01861\|IGHG4 | Immunoglobulin heavy constant gamma 4 | 35941 | 4.18 | 2 | 1.28 | 61.94 | 2 | 5.27 | |  |
| P01780\|HV307 | Immunoglobulin heavy variable 3-7 | 12943 | 1.78 | 1 | 1.37 | 34.67 | 2 | 1.77 | |  |
| P06312\|KV401 | Immunoglobulin kappa variable 4-1 | 13380 | 5.75 | 1 | 1.09 | 88.66 | 1 | 1.54 | |  |
| P01700\|LV147 | Immunoglobulin lambda variable 1-47 | 12284 | 7.77 | 1 | 1.07 | 17.39 | 1 | 1.99 | |  |
| P01709\|LV208 | Immunoglobulin lambda variable 2-8 | 12382 | 4.63 | 1 | 1.28 | 77.35 | 1 | 2.14 | |  |
| P01042\|KNG1 | Kininogen-1 | 71957 | 0.04 | 15 | 1.16 | 51.81 | 7 | 1.96 | |  |
| P80108\|PHLD | Phosphatidylinositol-glycan-specific phospholipase D | 92336 | 2.55 | 3 | 1.10 | 31.65 | 2 | 1.52 | |  |
| P02743\|SAMP | Serum amyloid P-component | 25387 | 2.23 | 8 | 0.93 | 58.39 | 7 | 1.82 | |  |
| P02766\|TTHY | Transthyretin | 15887 | 2.47 | 3 | 1.08 | 47.27 | 3 | 1.88 | |  |
| ***Down-regulated proteins in placebo cohort*** | | | | | | | | | |  |
| P15924\|DESP | Desmoplakin | 331774 | 1.13 | 2 | 1.34 | 200 | 3 | 0.37 | |  |
